# Influence of substrate formulation on some morphometric characters and biological efficiency of *Pleurotus ostreatus* EM‐1 (Ex. Fr) Kummer grown on rice wastes and “wawa” (*Triplochiton scleroxylon*) sawdust in Ghana

**DOI:** 10.1002/fsn3.2802

**Published:** 2022-03-21

**Authors:** Michael Wiafe‐Kwagyan, George Tawia Odamtten, Nii Korley Kortei

**Affiliations:** ^1^ 58835 Department of Plant and Environmental Biology College of Basic and Applied Sciences University of Ghana Legon Ghana; ^2^ Department of Nutrition and Dietetics School of Allied Health Sciences University of Health and Allied Sciences Ho Ghana

**Keywords:** amended and unamended rice wastes, biological efficiency, cap diameter, correlation, *P. ostreatus*, stipe length, supplemented substrates

## Abstract

A study was conducted to correlate the stipe length, cap diameter, and growth yield (fresh weight) of the fruiting body of *Pleurotus ostreatus* strain EM‐1 using different rice lignocellulosic wastes and “wawa” (*Triplochiton scleroxylon*) compost: raw unamended rice straw; rice straw amended with 1% CaCO_3_ and 10% CaCO_3_; rice straw amended with 1% CaCO_3_ and 10% CaCO_3_ supplemented with 5, 10, and 15% rice bran prior to bagging; rice straw and rice husk mixture (1:1 w/w) amended with 1% CaCO_3_ and 10% CaCO_3_ supplemented with 5%–15% rice bran prior to bagging; and wawa sawdust amended with 1% CaCO_3_ and 10% rice bran. The experiment was laid out in a completely randomized design in a well‐ventilated semi‐dark room at 26–28°C and 60%–65% ERH. The fresh weight, length of the stipe, and cap diameter increased differentially in each treatment with an increasing period of composting in the substrates. There was a good coefficient of determination (*R* ^2^) among stipe length, cap diameter, and biological efficiency (%). The *R* ^2^ among stipe length, cap diameter, and biological efficiency for the different formulated substrates ranged between *R* ^2^ = 0.6346–0.9454 and *R* ^2^ = 0.570–0.9624, respectively. The highest *R* ^2^ was obtained on raw unamended rice straw substrates (stipe length and cap diameter were *R* ^2^ = 0.9454 and *R* ^2^ = 0.9444, respectively), whereas the lowest correlation coefficient among stipe length, cap diameter, and biological efficiency (%) (*R* ^2^ = 0.6346; *R* ^2^ = 0.0570), respectively, was recorded on the rice straw and rice husk mixture substrates. The results show a positive correlation in morphometric growth parameters studied on the different formulated substrates.

## Graphical abstract

A study was conducted to correlate morphometric characters such as the stipe length, cap diameter, and growth yield (Biological Efficiency) of the fruiting body of *Pleurotus ostreatus* strain EM‐1 on different substrates formulations (i.e., rice wastes and *Triplochiton scleroxylon* sawdust) compost.

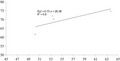

## 1 INTRODUCTION

The oyster mushrooms belong to the phylum Basidiomycota of the fungi. They are saprophytic in their nutrition, forming fruiting bodies (basidiocarps), fleshly in nature, and sometimes tough umbrella‐like sporophytes, which bear their spores on structures called basidia on the surface of the radiating gills (lamellae). Oyster mushrooms (*Pleurotus* spp.) are one of the most popularly cultivated mushrooms in the world with annual global production of over 900 metric tons (Sanchez, 2010). The ease of growing oyster mushrooms lies in the fact that *Pleurotus* spp. have the ability to grow at a wide temperature range utilizing various lignocellulose substrates by employing their ability to produce cellulosic and pectinolytic enzymes (Sanchez, 2010). The enzymatic efficiency of mushrooms and of their mode of nutrition makes them one of the most efficient organisms to employ in biotechnological solid‐state substrate fermentations. Mushrooms have the ability for converting cellulolytic and organic waste into palatable proteins food and medicinal nutraceuticals (Banik & Nandi, 2004; Chang, 2007; Gregori et al., 2007; Mahbuba et al., 2010; Mandeel et al., 2005; Zadrazil & Brunnert, 1981). The technology used for its cultivation is eco‐friendly since it exploits the natural ability of the fungus to degrade complex carbohydrates and polysaccharides to generate much simple compounds useful for human health and nutrition (Chang & Buswell, 1996).

The current state‐of‐the‐art research shows the usage of fungal biotechnology in other fields such as restoration of damage environments (mycorestoration), via mycofiltration (i.e., use of mycelia to filter water), mycoforestry (i.e., use of mycelia to restore forest), mycoremediation (i.e., use of mycelia to ameliorate heavily polluted soils), myconuclear bioremediation (i.e.,use of mycelia to sequester soil of radioactive materials), mycopesticide (used as biopesticide to control pests), and also spent compost could be used as biofertilizer to enhance the fertility of the soil (Adenipekun & Lawal, 2012; Kortei et al., 2018; Stamets, 2005; Wiafe‐ Kwagyan & Odamtten, 2018; Wiafe‐Kwagyan et al., 2016). These instances represent fungal ability to restore the ecosystem where there are no adverse effects after fungal application.

The use of different agricultural lignocellulosic waste for production of mushrooms has been studied extensively in many developed and developing countries. Agriculture wastes such as maize cob, maize stover, maize straw, soybeans, sugarcane bagasse, barley, wheat straw, and palm kernel have been used extensively for the cultivation of mushrooms (Chukwurah et al., 2013; Hòa et al., 2015; Howard et al., 2003; Kortei et al., 2018; Kortei & Wiafe‐Kwagyan, 2014; Wiafe‐Kwagyan et al., 2016). According to Chang (1993, 2006), cultivation of edible mushrooms has become an alternative economic venture even to date, mainly due to the increase in demand and increased market value as well as consumer appreciation of their delicacy, taste, high nutritional, and medicinal values. For example, there is the presence of high‐quality proteins, carbohydrates, and other polysaccharides, and mineral elements (e.g., Ca, P, Fe, K, Mg, Se, etc.), vitamins (thiamine, riboflavin, niacin, etc.) (Chang & Buswell, 1996; Kortei et al., 2017; Mattila et al., 2001), as well as low fat. Mushrooms such as *Pleurotus* spp. have therapeutic value in preventing diseases like hypertension, hypercholesterolemia, cancer, diabetes, as well as antibacterial and antiviral properties (Assan & Mpofu, 2014; Deepalakshmi & Mirunalini, 2014; Kortei et al., 2017; Odmtten, 2018; Wiafe‐Kwagyan et al., 2016).

In comparison to other edible mushrooms, *Pleurotus* species need a short growth time, and their fruiting bodies are not often attacked by diseases and insect pests (Hòa et al., 2015; Oseni et al., 2012; Wiafe‐Kwagyan et al., 2016). It is well known that different strains of *Pleurotus* respond differently to substrates used in their cultivation, the type of supplements, and the amounts of supplementation on the vigor of growth (Mahbuba et al., 2010; Visscher, 1989). Several studies suggest that the methods described for substrate preparation consist of composting agricultural residues followed by pasteurization in polypropylene bags (Kortei et al., 2017; Mandeel et al., 2005; Obodai et al., 2003; Raymond et al., 2013) which is achieved by several ways (Balasubramanya & Kathe, 1996); for example, Stamets (2005) included steam sterilization, and recently, gamma irradiation by Kortei et al. (2017).

The influence of substrate formulation on growth, development, and fruit formation and production by the mushroom can be assessed by several ways including weight of the fruiting body and numbers, stipe length, pileus (cap) width, stipe girth, and not excepting the biological efficiency of the mushroom produced on a particular substrate. Chukwurah et al. (2013) stated that changes in stipe length, pileus (cap) width, and stipe girth of *Pleurotus ostreatus* grown on two different agricultural wastes were high, while lower values were obtained from those grown in substrate composed of single agricultural waste. Biological efficiency was highest (97.7%) in substrates made of maize cob and palm kernel cake.

According to Fan et al. (2000), biological efficiency (B.E) is an expression of the bioconversion of dry substrate to fresh fruiting bodies and indicates the formulation ability of the fungus exploiting the substrate:

$$B.E\;=\frac{\text{Fresh}\;\text{weight}\;\text{of}\;\text{mushroom}\;\text{(g)}}{\text{Dry}\;\text{weight}\;\text{of}\;\text{substrate}\;\text{(g)}}\times 100\%$$

Kortei et al. (2017) recorded relatively higher (≤65%–98%) biological efficiency of *P. ostreatus* on various formulated wawa sawdust (*Triplochiton scleroxylon*) either steam sterilized or gamma irradiated, which agreed with the data of Garo and Girma (2016) who reported range 31.8%–146% from their study of responses of oyster mushroom (*P. ostreatus*) as influenced by different substrates in Ethiopia. Gitte et al. (2014) also reported high biological efficiency of milky mushroom (*Calocybe indica*) on different substrates which ranged from 51.57% to 146.3%. On the contrary, low biological efficiency (BE) values reported by Obodai et al., (2003) ranged from 61.0% to 0.0% on different lignocellulosic byproducts such as composted sawdust and elephant grass, respectively.

Varied degrees of growth of *P. ostreatus* fruit body parts have been observed on different substrates as well as different cultivation methods by some researchers (Grimm & Wösten, 2018; Nongthombam et al., 2021; Oseni et al., 2012; Sanchez, 2010). It is therefore possible to estimate the ability of mushrooms to convert substrates into edible mass for consumption. Indeed, Chukwurah et al. (2013), Garo and Girma (2016), and Gunde‐Cimerman and Cimerman (1995) showed that oyster mushrooms can have different morphometric dimensions (pileus/cap diameter, stipe length, stipe girth, and biological efficiency) depending on the nature of the substrate. The fruiting bodies of oyster mushrooms are expected by virtue of availability of nutrients to differ with respect to their morphometric length of stipe, girth of stipe, cap diameter, and growth yield (weight of mushroom) on different substrates (Chukwurah et al., 2013; Dubey et al., 2019; Kortei et al., 2018; Shah et al., 2004;).

This study was designed to show possible correlation of some morphometric parameters (cap/stipe diameter and stipe length) and biological efficiency in relation to the type of nutrient and amendment of rice waste substrate and sawdust in composted cultures.

## 2 MATERIALS AND METHODS

### 2.1 Preparation of pure culture

Pure cultures of *Pleurotus eous* raised on PDA for 7 days were obtained from the National Mushroom Mycelium Bank at the Food Research Institute, Ghana. Stock cultures were grown on slants of potato dextrose agar (PDA) in McCartney tubes and on Petri dishes. All media used were sterilized at 1.05 kg/cm^3^ pressure for 15 min at 121℃ and the remaining were kept in a refrigerator at 8°C. Each sterilized bottle containing grains was aseptically inoculated with one 3 cm of the 1‐week‐old culture grown on PDA. Spawns were incubated for 14–21 days without illumination in an electric incubator (Tuttlingten™ WTC Binder, Germany) at a temperature of 28°C until complete mycelia. Fully complete spawns were kept in refrigerator at 8°C.

### 2.2 Preparation of spawn

The spawns were prepared using a modification of the method of spawn preparation adopted by Narh et al. (2011), Obodai (1992), and Stamets and Chilton (1983). Sorghum (*Sorghum bicolor*) grains were used for the spawn preparation. The grains were separately washed and steeped in water overnight. They were then thoroughly washed separately with tap water to ensure that dust and other particles had been removed, drained, and tied in a wire mesh. The grains were steamed in an autoclave (Priorclave, Model PS/LAC/EH150, England) at 105°C for 45 min to ensure that the steamed grains were cooked but intact since broken grains are more prone to contamination. Thereafter, they were air dried to cool on a wooden frame with a wire mesh. To each grain, 3% (w/w) of calcium carbonate (CaCO_3_) was added and thoroughly mixed manually. The grains were sterilized in an autoclave at 121°C for 1 h.

### 2.3 Substrate preparation

The method adopted by Mondal et al. (2010) and Obodai et al. (2003) was used for all substrate preparations. Dry paddy rice straw was chopped into small pieces about 2–5 cm long (Wiafe‐Kwagyan, 2014). Exactly 10 kg was weighed and put into two metal barrels. The tanks were then filled with 432 L of tap water. It was soaked for 3 h, removed, and strained using raffia baskets. It was allowed to settle for about 45 min to drain excess water. Once the excess water was drained, a sample was squeezed in the hands; when no water exudes through the fingers and rather dampness it is assumed that the desired moisture has been achieved. The substrate was gathered into a heap and fermented for 0, 4, 8, and 12 days. The unfermented or uncomposted rice straw served as the 0 day substrate. The compost was turned every 4 days using a shovel to obtain a uniform composting and to avoid anaerobic fermentation in the middle portion of the heap compost. Each of the compost has a dimension of 48 × 76 × 100 cm. The following substrates were formulated using rice wastes (i.e., rice straw, husk, and rice bran) and sawdust.

1. Unamended rice straw (i.e., no addition of supplements, e.g., rice bran and CaCO_3_)
2. Rice straw amended with 1% CaCO_3_ and 10% rice bran prior to composting
3. Rice straw amended with 1% CaCO_3_ and 10% CaCO_3_ prior to composting and supplemented with 5, 10, or 15% rice bran prior to bagging
4. Rice straw and rice husk mixture (1:1 w/w) amended with 1% CaCO_3_ and 10% CaCO_3_ prior to composting and supplemented with 5%, 10%, or 15% rice bran prior to bagging
5. Wawa sawdust amended with 1% CaCO_3_ and 10% rice bran prior to composting

### 2.4 Bagging of compost and sterilization of bagged substrates

At the end of the composting process, each of the compost (0, 4, 8, and 12 days) was mixed thoroughly for several times. The mixture was then moistened with some amount of water till the moisture content was about 65%–70%. When this moisture is attained, a sample was taken and squeezed in the hands until no water exudes through the fingers and only some dampness is felt. This moisture content level was found to be optimum for oyster mushroom production (Khanna & Garcha, 1984). The substrate was packed into heat‐resistance polypropylene bags of dimensions 32.5 × 9.7 × 8.7 cm. Each bag was filled with 1 kg of the substrate and then compacted, and the mouth of the bag was pushed through a polyvinyl chloride (PVC) pipe of dimension 2.0 cm thick and 2.5 cm long, which was then pulled down and fastened with a rubber band. This served as a bottleneck into which cotton wool was inserted. The purpose of the PVC pipe was to provide an opening for inoculation and for gaseous exchange. The bags were labeled appropriately before sent to oil drums (tanks) and placed on metal stoves fueled with gas. The bags were steam sterilized for about 3 h at a temperature of about 100°C.

### 2.5 Inoculation and incubation of compost bags

After the bags were steam sterilized and allowed to cool, approximately 30–100 grains (3.5 g) of spawn were inoculated into each bag of weight of 1 kg under sterile conditions and were then shaken to disperse the spawns (seeds) for uniform distribution of the grains in the bag. The inoculated bags were transferred into an incubation room and were left for as long as mycelia will grow through the grains in the bag. The incubation period ranged from 30 to 70 days depending on the spawn vigor, substrate treatment, and climatic conditions. The ambient temperature of the incubation room was maintained at 25–31°C. During this period, the mycelial growth rate was measured every week until the entire content of the bag was filled with the mycelia. After the spawn run period (i.e., the period required for complete impregnation of the substrate), the bags were transferred into a cropping house for the formation of fruit bodies once the mycelia become thickened in the bags.

### 2.6 Calculation of growth and yield parameters of mushroom

$$B\text{.E}=\frac{\text{Fresh}\;\text{weight}\;\text{of}\;\text{mushroom}}{\text{Dry}\;\text{weight}\;\text{of}\;\text{substrate}}\times 100\%$$

B.E, that is, the productivity of the conversion of substrate/lignocellulosic wastes into fruiting body (Chang et al. 1981). The fruit bodies were weighed immediately after harvesting using electronic weighing balance

Stipelength=lengthofcapbasetoendofstalk

Stipe length was measured by placing a string from one end, where it was attached to the substrate, to the point where the gills on the pileus start. The thread was placed on a 30 cm ruler for actual measurement values.

$$\text{Average}\;\text{cap}\;\text{diameter}=\frac{\text{longest}+\text{shortest}\;\text{cap}\;\text{diameter}}{2}$$

Cap diameter was measured by using a string passing from one end of the pileus to the other through the center of the pileus. The thread was placed on a 30 cm ruler for actual measurement values.

Yield(g/kg)=weightoffreshmushrooms

Yield of mushroom was determined as the total weight of all the fruiting bodies harvested from all the three flushes/pickings.

### 2.7 Statistical analysis

Linear regression analysis was employed using Excel for Microsoft 365 (Windows 10 version). Means of yield and growth parameters were subjected to analyses of variance (one‐way ANOVA). Differences between means were determined using Fisher's least significant difference (LSD) at alpha (LSD α ≤ .05). Values recorded were means with standard errors of five replicates.

## 3 RESULTS

### 3.1 Unamended rice straw only

**FIGURE 1:**
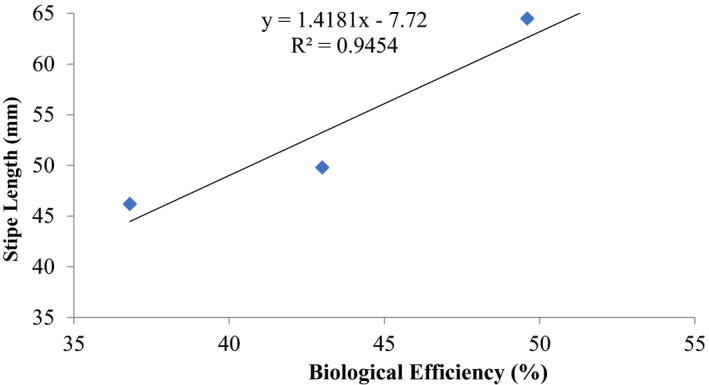
Correlation between biological efficiency and stipe length (mm) of *P. ostreatus* grown on unamended rice straw

**FIGURE 2:**
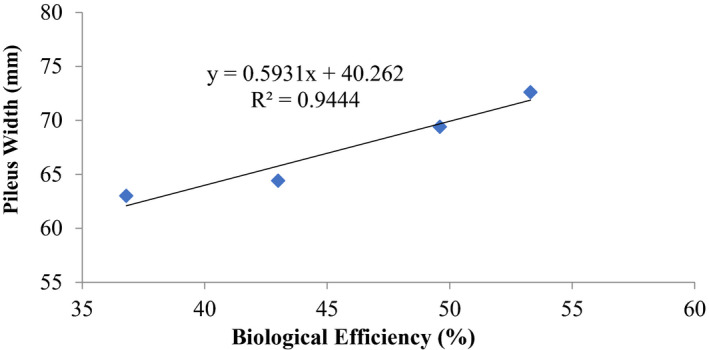
Correlation between biological efficiency and pileus width (mm) of *P. ostreatus* grown on rice straw only

The mean fresh weight of the mushroom (yield) and mean length of the stipe and cap (pileus) increased proportionately with increasing period of composting up to 12 days (Table 1). Mean weight of the fruiting body ranged from an initial 9.5 ± 2.0 g for the unamended substrate to 13.7 ± 2.1 g after 12 days. Cap diameter varied from 63.0 ± 3.5 mm to 67.7 ± 2.0 mm (Table 1). Correspondingly, stipe length varied from 46.2 ± 5.3 mm to 72.6 ± 3.1 mm. Coefficient of determination between stipe length and biological efficiency was *R* ^2^ = 0.945; the parallel value for cap diameter and biological efficiency was also positive *R* ^2^ = 0.944 (Figures 1 and 2). The observations made were statistically significant (*p* ≤ .05).

**TABLE 1:** Record of the morphometric data of mean diameter of cap, mean length of stipe, and mean weight of fruiting bodies of *P. ostreatus* EM‐1 grown on unamended rice straw during the indicated fermentation periods at 25–31°C

Period of composting (days)	Mean fresh weight (yield) of mushroom (g) ± SE	Mean diameter (mm) of cap ± SE	Mean length (mm) of stipe ± SE
0	9.5 ± 2.2^a^	63.0 ± 3.5^c^	46.2 ± 5.3^d^
4	12.4 ± 1.6^b^	64.4 ± 5.0^c^	49.8 ± 4.8^d^
8	12.8 ± 1.7^b^	64.5 ± 2.7^c^	69.4 ± 3.5^e^
12	13.7 ± 2.1^b^	67.7 ± 2.0^c^	72.6 ± 3.1^e^

Note

Values in the same column followed by a different letter are statistically significant (*p* ≤ .05) from each other.

### 3.2 Rice straw amended with 1% CaCO_3_ and 10% rice bran (RB)

The mean fresh weight of the fruiting body (yield), mean diameter of cap, and mean length of stipe increased up to 8 days composting and thereafter declined. The highest mean weight of the fruiting body was 16.7 ± 1.7 g after 8 days. The widest mean diameter of the cap 86.6 ± 8.1 mm was attained in 8 days composting, while the longest mean length of stipe 63.4 ± 7.9 was on the same substrate (Table 2).

**TABLE 2:** Record of the morphometric data of mean diameter of cap, mean length of stipe, and mean weight of fruiting bodies of *P. ostreatus* EM‐1 grown on rice straw amended with 1% CaCO_3_ and 10% rice bran during the indicated fermentation periods at 25–31°C

Period of composting (days)	Mean fresh weight (yield) of mushroom (g) ± SE	Mean diameter (mm) of cap ± SE	Mean length (mm) of stipe ± SE
0	10.8 ± 1.9^a^	61.1 ± 13.4^a^	43.6 ± 3.6^a^
4	14.1 ± 1.6^b^	62.3 ± 8.7^a^	51.4 ± 4.7^b^
8	16.7 ± 1.7^c^	86.6 ± 8.1^b^	63.4 ± 7.9^c^
12	14.3 ± 1.8^b^	77.3 ± 5.8^c^	58.5 ± 6.6^c^

Note

Values in the same column followed by a different letter are statistically significant (*p* ≤ .05) from each other.

**FIGURE 3:**
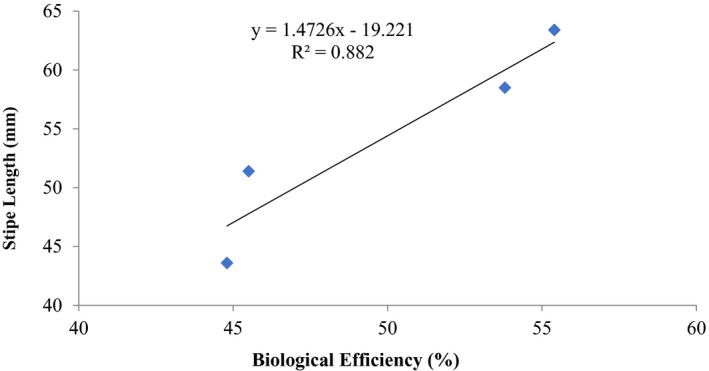
Correlation between biological efficiency and stipe length (mm) of *P. ostreatus* grown on rice straw amended with 1% CaCO_3_ and 10% rice bran

**FIGURE 4:**
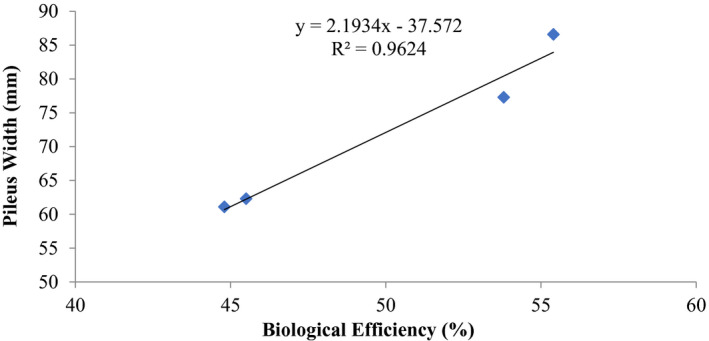
Correlation between biological efficiency and pileus width (mm) of *P. ostreatus* grown on rice straw amended with 1% CaCO_3_ and 10% rice bran

The correlation relationships between stipe length and cap diameter of the fruiting bodies with biological efficiency are summarized in Figures 3 and 4. There was a good positive correlation coefficient between these morphometric measurements and biological efficiency. The coefficient of determination between stipe length and biological efficiency was *R* ^2^ = 0.882 (Figure 3), while that of diameter of cap with biological efficiency was *R* ^2^ = 0.9624 (Figure 4).

### 3.3 Rice straw amended with 1% CaCO_3_ and 10% rice bran supplemented with additional 5, 10, or 15% rice bran prior to bagging

Generally, the mean fresh weight (yield), stipe length, and cap diameter increased in some instances but was distinct up to 4 days of composting. Supplementation with rice bran did not seem to increase yield significantly (*p* ≤ .05) at least up to 4 days of composting. The highest yield of fresh weight of the fruiting bodies was in the bags amended with 5% rice bran (20.2 ± 6.7g) and composted for 8 days, followed by 12 days composted substrate amended with 10% rice bran (18.2 ± 6.4 g) (Table 3). The corresponding highest diameter of cap (81.2 ± 13.4 mm) was also recorded in the 8 days composted substrate supplemented with 5% rice bran (Table 3), followed in a similar manner by the fruiting bodies produced on the 12‐day composted substrate amended with 10% rice bran (76.3 ± 4.9 mm). The mean length of the stipe followed the same trend (Table 3).

**TABLE 3:** Record of the morphometric data of mean diameter of cap, mean length of stipe, and mean weight of fruiting bodies of *P. ostreatus* EM‐1 grown on rice straw amended with 1% CaCO_3_ and 10% CaCO_3_ supplemented with 5, 10, or 15% rice bran prior to bagging during the indicated fermentation periods at 25–31°C

Period of composting (days)	Rice bran added (%)	Mean fresh weight (yield) of mushroom (g) ± SE	Mean diameter (mm) of cap ± SE	Mean length (mm) of stipe ± SE
0	5	9.7 ± 1.9^a^	42.7 ± 6.7^a^	44.0 ± 6.8^a^
	10	10.3 ± 2.8^a^	43.0 ± 3.7^a^	46.4 ± 5.7^a^
	15	11.1 ± 2.7^a^	45.6 ± 4.7^a^	54.2 ± 10.2^b^
4	5	12.5 ± 3.2^a^	47.8 ± 5.2^a^	60.8 ± 7.8^c^
	10	13.4 ± 3.5^a^	53.1 ± 7.8^b^	63.2 ± 9.9^c^
	15	13.9 ± 2.1^a^	53.4 ± 1.8^b^	63.5 ± 11.9^c^
8	5	20.2 ± 6.7^b^	74.0 ± 8.2^c^	81.2 ± 13.4^d^
	10	15.5 ± 3.0^c^	56.4 ± 9.5^b^	68.9 ± 4.7^c^
	15	16.1 ± 3.4^c^	57.3 ± 7.1^b^	69.0 ± 10.3^e^
12	5	12.7 ± 2.6^a^	52.4 ± 7.1^b^	60.2 ± 2.9^c^
	10	18.2 ± 6.4^d^	62.0 ± 10.5^c^	76.3 ± 4.9^d^
	15	14.5 ± 2.6^c^	55.5 ± 10.5^b^	67.2 ± 13.2^e^

Note

Values in the same vertical column followed by same letter do not differ significantly (*p* ≤ .05) from each other.

**FIGURE 5:**
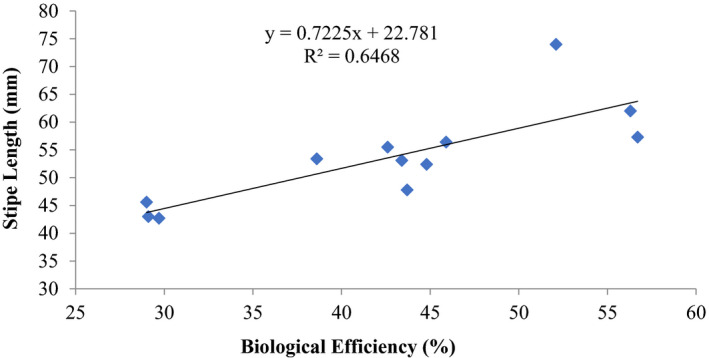
Correlation of stipe length (mm) and biological efficiency of *P. ostreatus* grown on rice straw amended with 1% CaCO_3_ and 10% rice bran at composting supplemented with different proportions (5, 10, 15) % of rice bran prior to bagging

**FIGURE 6:**
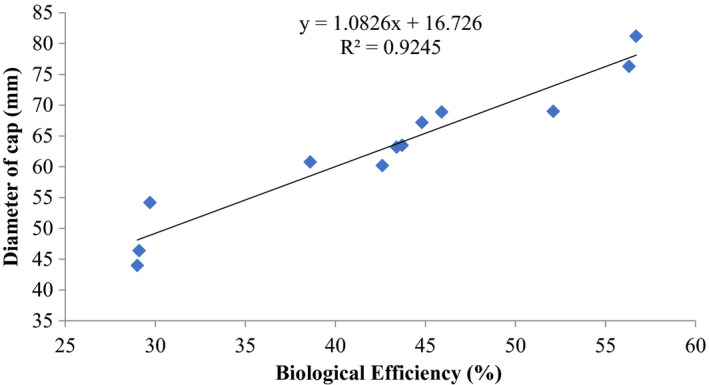
Correlation of cap diameter (mm) and biological efficiency of *P. ostreatus* grown on rice straw amended with 1% CaCO_3_ and 10% rice bran at composting and supplemented with different proportions (5, 10, 15) % of rice bran prior to bagging

There was also a good positive coefficient of determination *R* ^2^ = 0.6468 (stipe length) and *R* ^2^ = 0.9245 (cap diameter) and biological efficiencies of *P. ostreatus* (Figures 5 and 6).

### 3.4 Rice straw and rice husk mixture (1:1 w/w) amended with 1% CaCO_3_ and 10% rice bran and supplemented with 5, 10, or 15% rice bran prior to bagging

The growth of morphometric parameters (weights/yield, cap diameter, and stipe length) changed marginally or was insignificant (*p* > .05) within the same treatment irrespective of the period of composting and supplementation (Table 4). The widest cap diameter was recorded on substrates supplemented with 5%–10% rice bran and composted for 12 days; correspondingly, the longest stipe was also obtained on the medium supplemented with 5%–10% rice bran and composted for 12 days (Table 4).

**TABLE 4:** Record of the morphometric data of mean diameter of cap, mean length of stipe, and mean weight of fruiting bodies of *P. ostreatus* EM‐1 grown on rice straw and rice husk mixture (1:1 w/w) amended with 1% CaCO_3_ and 10% CaCO_3_ and supplemented with 5, 10, or 15% rice bran prior to bagging during the indicated fermentation periods at 25–31°C

Period of composting (days)	Rice straw/rice husk mixture (1:1 w/w) amended with rice bran (%)	Mean fresh weight (yield) of mushroom (g) ± SE	Mean diameter (mm) of cap ± SE	Mean length (mm) of stipe ± SE
0	5	12.0 ± 2.6^a^	50.0 ± 8.0^a^	54.0 ± 5.0^a^
	10	12.4 ± 2.6^a^	47.0 ± 4.0^a^	54.0 ± 6.0^a^
	15	13.4 ± 3.5^a^	42.0 ± 8.0^b^	50.0 ± 3.0^a^
4	5	13.5 ± 3.7^a^	52.0 ± 10.0^a^	65.0 ± 5.0^b^
	10	14.8 ± 2.2^a^	55.0 ± 5.0^a^	63.0 ± 8.0^b^
	15	9.9 ± 1.8^b^	60.0 ± 6.0^c^	61.0 ± 12.0^b^
8	5	14.9 ± 2.6^a^	53.0 ± 8.0^a^	63.0 ± 8.0^b^
	10	13.3 ± 2.2^a^	52.0 ± 11.0^a^	59.0 ± 6.0^c^
	15	10.3 ± 2.5^c^	53.0 ± 8.0^a^	64.0 ± 9.0^b^
12	5	13.7 ± 2.2^a^	65.0 ± 12.0^c^	68.0 ± 14.0^b^
	10	14.6 ± 3.7^a^	66.0 ± 9.0^c^	70.0 ± 6.0^d^
	15	13.8 ± 2.8^a^	66.0 ± 8.0^c^	67.0 ± 9.0^b^

Note

Values in the same vertical column followed by same letter do not differ significantly (*p* ≤ .05) from each other.

**FIGURE 7:**
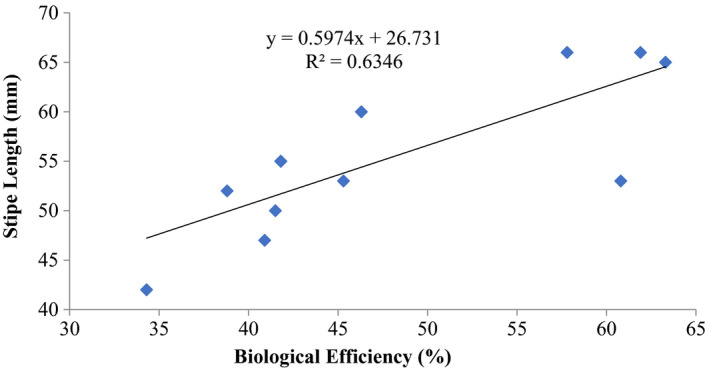
Correlation of stipe length (mm) and biological efficiency of *P. ostreatus* grown on rice straw and rice husk mixture (1:1 w/w) amended with 1% CaCO_3_ and 10% rice bran and supplemented with different proportions (5, 10, 15) % of rice bran prior to bagging

**FIGURE 8:**
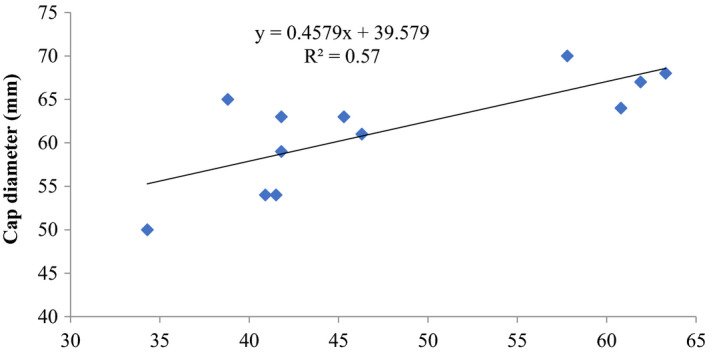
Correlation of cap diameter (mm) and biological efficiency of *P. ostreatus* grown on rice straw and rice husk mixture (1:1 w/w) amended with 1% CaCO_3_ and 10% rice bran supplemented with different proportions (5, 10, 15) % of rice bran prior to bagging

The correlations between biological efficiencies (%) and stipe length (mm) as well as cap diameter (mm) of *P. ostreatus* showed that there was a positive and moderate coefficient of determination *R* ^2^ = 0.63345 (stipe length) and *R* ^2^ = 0.57 (cap diameter) and biological efficiencies, and for *P. ostreatus* from this substrate formulations (Figures 7 and 8).

### 3.5 Wawa sawdust amended with 1% CaCO_3_ and 10% rice bran and composted for up to 12 days

**FIGURE 9:**
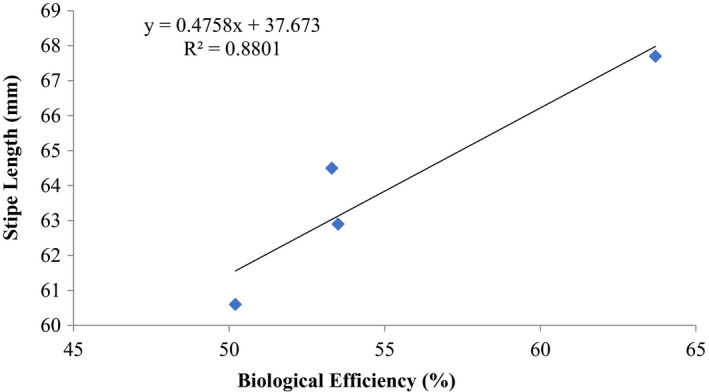
Correlation between biological Efficiency and stipe Length (mm) of *P. ostreatus* grown on ‘wawa’ sawdust amended with 1% CaCO_3_ and 10% rice bran at 25–31°C

**FIGURE 10:**
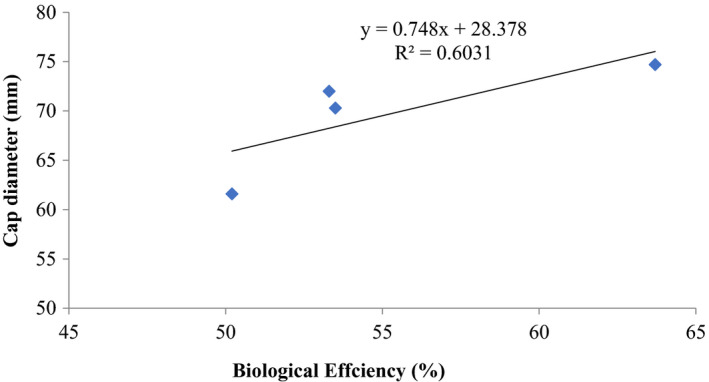
Correlation between biological efficiency and cap diameter (mm) of *P. ostreatus* grown on ‘wawa’ sawdust amended with 1% CaCO_3_ and 10% rice bran at 25–31°C

Table 5 and Figures 9 and 10 summarize results obtained. The fresh weight, diameter of cap, and the length of stipe of the fruiting bodies obtained from the compost increased with increasing time of composting up to 8 days, and thereafter declined (Table 5).

**TABLE 5:** Correlation among fresh weight of mushroom, stipe length, and cap diameter of *P. ostreatus* EM‐1 grown on wawa sawdust amended with 1% CaCO_3_ and 10% rice bran during the indicated fermentation periods at 25–31°C

Period of composting (days)	Mean fresh weight (yield) of mushroom (g) ± SE	Mean diameter (mm) of cap ± SE	Mean length (mm) of stipe ± SE
0	7.4 ± 1.0^c^	61.6 ± 2.6^a^	60.6 ± 2.6^e^
4	7.6 ± 0.9^c^	70.3 ± 3.7^e^	62.9 ± 3.5^a^
8	13.1 ± 1.2^d^	74.7 ± 5.7^e^	67.7 ± 2.0^b^
12	9.9 ± 1.2^c^	72.0 ± 6.5^ce^	64.5 ± 2.7^a^

Note

Values in the same column followed by the same letter do not differ significantly (*p* ≤ .05) from each other.

The correlation between biological efficiencies (%) and stipe length (mm) as well as cap diameter (mm) of *P. ostreatus* showed a positive coefficient of determination of *R* ^2^ = 0.8801 (stipe length) and *R* ^2^ = 0.6031 (cap diameter) and their biological efficiencies (Figures 9 and 10).

## 4 DISCUSSION

The fruiting bodies of oyster mushrooms differ with respect to stipe length and girth, and pileus (cap) diameter when grown in different farm substrates (Garo & Girma, 2016; Shah et al., 2004). The results presented here showed that the changes in stipe length and cap diameter grown in different formulations of substrates depended on the constituents and that substrates yielded mushrooms of different sizes and weight (Table 1, 2, 3, 4, 5). The growth parameters of cap diameter and stipe length of *P. ostreatus* obtained on the various treatments in the formulation of the substrates varied presumably due to the extent of the release of nutrients from depolymerization of substrate (during composting) and subsequent mobilization of hyphae in the substrate for growth. The performance of oyster mushroom grown on composted lignocellulose substrates also depended on the structure, compactness, and physical properties of the substrate as well as the type of formulation and method used (Kortei et al., 2017). Chukwurah et al. (2013) reported that substrate with higher moisture capacity grow better than those of lower moisture. Substrate which contained mixture of different types of agricultural waste also performed better than those of single agricultural waste (Chukwurah et al., 2013). The moisture contents of the substrates used for the present experiments ranged from 65% to 70%, which fall within the range presented by Buswell (1984).

Our results of stipe lengths and cap diameters obtained in this experiment agree with the findings of other investigators (Chukwurah et al., 2013; Kortei et al., 2018; Obodai et al., 2003). On the other hand, Kortei and Wiafe‐Kwagyan (2014) reported higher values of 95 and 85 mm for cap diameter and stipe lengths, respectively, for *P.  eous* P‐31 strains cultivated on gamma‐irradiated corn cobs when they investigated the growth of P‐31 strain on eight different gamma‐irradiated substrates. Lower ranges of stipe length (1–5 cm) and 1.85–6.57 cm for cap diameter have been recorded by Kortei et al. (2018) and Sarker et al. (2007). These discrepancies observed in the present study and the others could be attributed to the type of substrates and different pre‐ and posttreatments used in the formulation of these substrates. It is well‐established that different strains of *Pleurotus* respond differently to substrates used in their cultivation (Mahbuba et al., 2010; Visscher, 1989). Furthermore, the mixing of substrates may help in the optimization of the compositional characters such as water holding capacity, increased substrate structure, and porosity (Hòa et al., 2015; Muswati et al., 2021). These qualities enhance water penetration and gaseous exchange, and an optimum C: N ratio that improves the efficiency of the substrates thereby helping to increase yield and morphometric characteristics such as biological efficiency, cap diameter, and stipe length (Hòa et al., 2015; Muswati et al., 2021).

In a similar investigation by Dubey et al. (2019), the highest stipe length was obtained in rice straw 4.8575 cm, followed by banana leaves (4.3425 cm), wheat straw (3.675 cm), and lastly, sugarcane (3.275 cm). Likewise, cap diameter was also found highest for rice straw, that is, 5.135 cm, followed by wheat straw, banana leaves, and lastly, sugarcane bagasse, which were 4.105 cm, 3.48 cm, and 3.255 cm, respectively, under similar environment and cultural practices among other substrates.

Other previous workers (e.g., Oseni et al., 2012; Sanchez, 2010) have reported varied growth of *P. ostreatus* fruit body parts on different substrates as well as different cultivation methods. In this study, there was high coefficient of correlation of stipe length and biological efficiency (0.646%–0.945%) similar to cap diameter and biological efficiency (0.579%–0.962%) (Figures 1, 2, 3, 4, 5, 6, 7, 8, 9, 10).

The highest coefficient of regression line between biological efficiency and stipe length and cap diameter was obtained in Figures 1, 2, 3, 4, 6, and 8. The more scattered points were obtained for complex media formulation (Figures 5, 6, 7, 8) with low *R* ^2^. In the case of yield, the larger the cap diameter, the higher the yield. Fruiting body weight is to a large extent influenced by the thickness and diameter of the cap. Large‐sized fruit bodies are widely perceived to be of superior quality, and hence highly ranked in mushroom cultivation in estimating pricing.

## 5 CONCLUSION

Mushrooms are important due to their nutritive and medicinal values (Agrahor‐Murugkar & Subbulakshmi, 2005; Cheung & Cheung, 2005). *Pleurotus ostreatus* grows in tropical and subtropical rainforest but can be cultivated artificially. It has high levels of proteins, carbohydrates, minerals (calcium, phosphorus, iron, potassium, magnesium, sodium, zinc, lead, etc.) (Mattila et al., 2001; Patil et al., 2010; Wiafe‐Kwagyan, 2014), and vitamins (thiamin, riboflavin, and niacin), as well as low fat (Hòa et al., 2015; Manzi et al., 1999). The fresh mushroom contains 85%–90% moisture, 3% protein, 4% carbohydrates, 0.3%–4.0% fat, and vitamins. Consumption patterns of mushrooms, particularly *P. ostreatus* in Ghana, have increased because of its ease of cultivation on artificial media, which has been acknowledged as a functional food. The trading in mushrooms, however, requires standardization of quality. In grading oyster mushrooms for pricing (Kortei et al., 2018; Onyango et al., 2011) have suggested the use of correlation of stipe length, cap diameter (pileus width), and weight of fruiting body as good criteria in grading quality. Our present results agree with this viewpoint. There was a good positive correlation among cap diameters, stipe lengths, and biological efficiency on all substrates used. But the morphometric values and B.E were commensurate with the type of five nutrients and amendments of rice waste and composted sawdust. The results show a reliable indication of the possibility to predict mushroom yield and biological efficiency with these growth determinants.

## AUTHOR CONTRIBUTIONS

**Michael Wiafe‐Kwagyan:** Conceptualization (equal); Methodology (equal); Supervision (equal); Writing – original draft (equal); Writing – review & editing (equal). **George Tawia Odamtten:** Conceptualization (equal); Supervision (equal); Writing – original draft (equal). **Nii Korley Kortei:** Data curation (equal); Software (equal); Validation (equal); Writing – review & editing (equal).

## Data Availability

As the corresponding author, I take full responsibility for the integrity of the data and the accuracy of the data analysis. Any other data related to the manuscript would be made available upon request.
